# Nutrient Deficiency and an Algicidal Bacterium Improved the Lipid Profiles of a Novel Promising Oleaginous Dinoflagellate, *Prorocentrum donghaiense,* for Biodiesel Production

**DOI:** 10.1128/AEM.01159-21

**Published:** 2021-09-10

**Authors:** Jiali Gui, Shuangshuang Chen, Guiying Luo, Zixiang Wu, Yongxiang Fan, Luming Yao, Hong Xu

**Affiliations:** a State Key Laboratory of Cellular Stress Biology, School of Life Sciences, Xiamen University, Xiamen, Fujian, People’s Republic of China; b Key Laboratory of the Ministry of Education for Coastal and Wetland Ecosystems, Xiamen University, Xiamen, Fujian, People’s Republic of China; University of Illinois at Urbana—Champaign

**Keywords:** *Prorocentrum donghaiense*, lipid accumulation, nutrient starvation, algicidal bacteria, DHA, biodiesel production

## Abstract

The lipid production potentials of 8 microalgal species were investigated. Among these 8 species, the best strain was a dominant bloom-causing dinoflagellate, Prorocentrum donghaiense; this species had a lipid content of 49.32% ± 1.99% and exhibited a lipid productivity of 95.47 ± 0.99 mg liter^−1^ day^−1^, which was 2-fold higher than the corresponding values obtained for the oleaginous microalgae Nannochloropsis gaditana and Phaeodactylum tricornutum. *P. donghaiense,* which is enriched in C_16:0_ and C_22:6_, is appropriate for commercial docosahexaenoic acid (DHA) production. Nitrogen or phosphorus stress markedly induced lipid accumulation to levels surpassing 75% of the dry weight, increased the C_18:0_ and C_17:1_ contents, and decreased the C_18:5_ and C_22:6_ contents, and these effects resulted in decreases in the unsaturated fatty acid levels and changes in the lipid properties of *P. donghaiense* such that the species met the biodiesel specification standards. Compared with the results obtained under N-deficient conditions, the enhancement in the activity of alkaline phosphatase of *P. donghaiense* observed under P-deficient conditions partly alleviated the adverse effects on the photosynthetic system exerted by P deficiency to induce the production of more carbohydrates for lipogenesis. The supernatant of the algicidal bacterium *Paracoccus* sp. strain Y42 culture lysed *P. donghaiense* without decreasing its lipid content, which resulted in facilitation of the downstream oil extraction process and energy savings through the lysis of algal cells. The Y42 supernatant treatment improved the lipid profiles of algal cells by increasing their C_16:0_, C_18:0_, and C_18:1_ contents and decreasing their C_18:5_ and C_22:6_ contents, which is favorable for biodiesel production.

**IMPORTANCE** This study demonstrates the high potential of Prorocentrum donghaiense, a dominant bloom-causing dinoflagellate, for lipid production. Compared with previously studied oleaginous microalgae, *P. donghaiense* exhibit greater potential for practical application due to its higher biomass and lipid contents. Nutrient deficiency and the algicidal bacterium *Paracoccus* sp. strain Y42 improved the suitability of the lipid profile of *P. donghaiense* for biodiesel production. Furthermore, *Paracoccus* sp. Y42 effectively lysed algal cells, which facilitates the downstream oil extraction process for biodiesel production and results in energy savings through the lysing of algal cells. This study provides a more promising candidate for the production of docosahexaenoic acid (DHA) for human nutritional products and of microalgal biofuel as well as a more cost-effective method for breaking algal cells. The high lipid productivity of *P. donghaiense* and algal cell lysis by algicidal bacteria contribute to reductions in the production cost of microalgal oil.

## INTRODUCTION

The growth of the global population has led to increasing demand for the resources needed in daily life, of which, food and energy are the most important. In past decades, microalgae have attracted substantial attention for diverse purposes, such as the extraction of nutritional and medicinal compounds and other important bioproducts, due to their high contents of cellular lipids, proteins, carbohydrates, polysaccharides, pigments, vitamins, and bioactive substances (1–3). Microalgae are considered natural cell factories of bioactive compounds that can be used in different biological applications. The world energy crisis in the 1970s made it imperative to exploit and utilize renewable energy. During combustion, biofuels such as biodiesel and bioethanol produce less carbon monoxide, particulate matter, hydrocarbon, and sulfur dioxide than petrodiesel. Therefore, biofuel has become a promising, environmentally friendly, alternative energy source for global fuel markets. Microalgae are a promising, easy-to-cultivate, sustainable biofuel source with a high growth rate and high lipid production within a short life cycle; therefore, they have great potential for large-scale production (4, 5). Moreover, in addition to nutritional and medicinal applications, microalgal fatty acids are renewable and sustainable sources for biofuel production.

In the field of microalgal biofuels, lipid productivity takes into account the lipid content within cells and the biomass produced by cells and serves as the standard for assessing the biofuel potential of a species (6, 7). Therefore, the most important parameters when screening microalgal strains for biodiesel production are a high lipid content and high biomass productivity (8). Chlorophyta, Chrysophyta and Bacillariophyta are the most widely studied microalgae for biofuel production. The lipid content of *Chlamydomonas* (9, 10), *Dunaliella* (11), *Nannochloropsis* (12, 13), *Chlorella* (14), *Tribonema* (15), *Hormidium* (15), *Zygnema* (15), *Scenedesmus* (16), *Tetraselmis* (17), *Phaeodactylum* (18, 19), *Isochrysis* (19), *Porphyridium* (20), and *Schizochytrium* (21) species can reach up to 20 to 50% of their dry weight. Several *Nannochloropsis* species are considered industrial microalgae because they produce larger amounts of lipids, at 37 to 60% of the dry weight (5, 22). However, the current production cost of microalgal oil would need to be reduced 10 times for microalgal oil to become competitive with fossil fuel oil (23). Nutrient, temperature, and chemical stresses are effective means for triggering oil accumulation in algae (24–27). However, these approaches reduce photosynthesis and, thus, the overall production of biomass (25). Detailed studies on strain selection, optimization of culture conditions, screening of chemical triggers, large-scale bioreactor development, bioengineering or targeted manipulation of certain enzymes for better biomass and biofuel production, and improvements in biomass harvesting and other downstream processing are being carried out to reduce production costs (25, 27–29). To make microalgal fuels for power generation economically viable, screening for microalgal strains that have the potential to achieve more efficient lipid production is crucial. The marine microalga Parachlorella kessleri was recently reported to be a novel highly efficient lipid producer with better lipid productivity and a higher lipid content than other microalgae, at approximately 40% to 60% of dry weight (7, 30). Dinoflagellates are important phytoplankton and are widely distributed. Most of these organisms can grow and produce large blooms under natural conditions. These blooms can extend for hundreds of kilometers, with cell abundances of millions of cells per liter (31). Certain species of dinoflagellates have been studied for decades, because they are associated in some cases with toxic and noxious events, such as massive mortality events for different marine organisms and serious threats to human health. However, their potential use for the production of bioactive substances or biofuels is poorly known. Only Alexandrium minutum (32) and Karlodinium veneficum (32, 33) have been reported to accumulate lipids to >20% of their dry weight.

Algal biodiesel has attracted broad public interest and large investment owing to its advantages, such as environmental friendliness, renewability, and sustainability. However, it is difficult to commercialize algal biodiesel because the current technology to produce conventionally usable fuel from algae requires numerous conversion steps. The most noteworthy part of the process is the disruption of the cells in the downstream process of lipid extraction. Since microalgal lipids are usually wrapped in the cell wall of the algal body, the cell wall-breaking treatment is an important pretreatment step for effective lipid extraction. Selecting the appropriate cell lysis method is particularly important for the application of biodiesel, as these processes can account for up to 26.2% of the energy input during lipid extraction (34). Typically, the cell disruption techniques applied to microalgae are classified as mechanical or nonmechanical cell breaking. Biolysis methods have the advantages of low energy consumption and the potential for development; therefore, they have gradually attracted attention. However, the majority of reports about algicidal bacteria have focused on bacteria acting against bloom-forming algae (35). Some of the bioactive molecules produced by bacteria are capable of provoking microalgal hydrolysis, thereby causing growth inhibition, death, and complete algal disruption. Furthermore, the relationship between bacteria and microalgae has been highlighted recently as a potential strategy to be applied in biofuel production processes. The disruption of microalgae by algicidal bacteria as a biolysis treatment for biofuel production processes has been reported. It has been found that the cell wall of microalgae can be damaged if an oil-rich microalga (Chlorella vulgaris ESP-1) is cocultured with the indigenous bacterial isolate Flammeovirga yaeyamensis in a salt concentration of 3% and a pH of 8.0. A nearly 100% increase in lipid extraction efficiency was obtained (36). Two strains of bacteria, Pseudomonas pseudoalcaligenes AD6 and Aeromonas hydrophila AD9, were identified and demonstrated to exhibit algicidal activity against the microalgae Neochloris oleoabundans and Dunaliella tertiolecta. Aeromonas hydrophila AD9 showed a nearly 12-fold increase in lipid extraction with *D. tertiolecta*, while both bacteria showed a 6-fold improvement in lipid extraction with *N. oleoabundans* (35). However, the effect of algicidal bacteria on microalgal lipid composition has been less reported.

In this study, we investigated the lipid contents of six harmful algal bloom (HAB)-causing dinoflagellate species and compared their lipid productivity with that of the oleaginous microalgae Phaeodactylum tricornutum and Nannochloropsis gaditana. Prorocentrum donghaiense, a dominant, nontoxic bloom-forming dinoflagellate in the Yangtze River Estuary and the adjacent East China Sea that has caused frequent, large-scale algal blooms and usually results in serious damage to marine ecosystems and mariculture, leading to enormous economic losses over the past 2 decades (37), was selected for further evaluation of its fatty acid profiles and lipid production potential under nutrient deficiency due to its high lipid contents. Moreover, an algicidal bacterium, *Paracoccus* sp. strain Y42, was used not only to lyse algal cells to facilitate the downstream extraction process but also to improve the lipid composition of the microalgal cells to make them more favorable for biodiesel production.

## RESULTS AND DISCUSSION

### Growth and lipid accumulation properties of 8 microalgal species.

**FIG 1:**
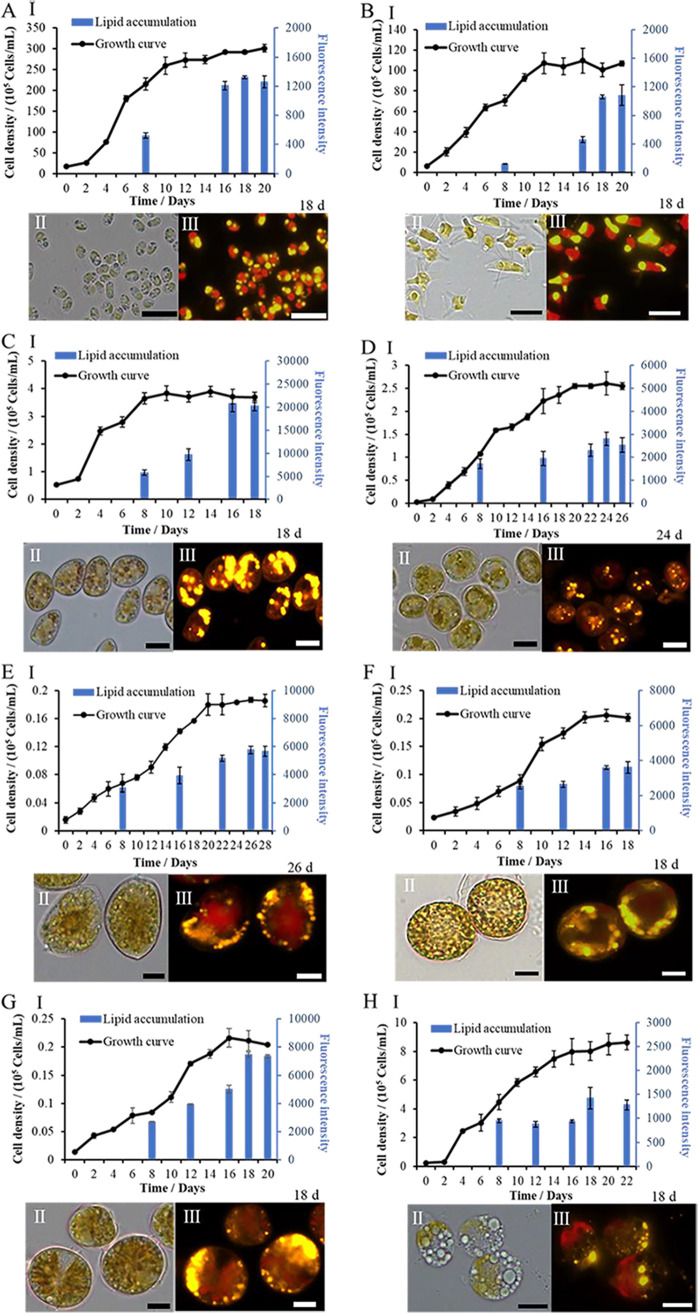
Growth and lipid accumulation of *Nannochloropsis gaditana* (A), *Phaeodactylum tricornutum* (B), *Prorocentrum donghaiense* (C), *Prorocentrum minimum* (D), *Prorocentrum micans* (E), *Scrippsiella trochoidea* (F), *Alexandrium minutum* (G), and Amphidinium carterae (H). (I) Growth and lipid accumulation of algal cells. (II) Photographs of algal cells. (III) Lipid droplets were stained with Nile red and observed with a fluorescence microscope. Bars, 10 μm.

To evaluate the ability of marine dinoflagellates to produce lipids, their growth and lipid accumulation were measured. As shown in Table 1 and Fig. 1, five dinoflagellate species (*A. minutum*, Amphidinium carterae, *P. donghaiense*, Prorocentrum micans, and Scrippsiella trochoidea) in this study showed remarkable abilities to accumulate lipids, surpassing 20% of dry weight under the same cultivation conditions. In particular, *P. donghai*ense exhibited the highest lipid content at 49.32% ± 1.99% dry weight, which was higher than those of the oleaginous microalgae *N. gaditana* (41.39% ± 3.95%) and *P. tricornutum* (33.58 ± 3.07%). In addition to lipid content, the potential of algae for lipid production also relies on their growth and biomass production. Therefore, it is necessary to further evaluate the lipid productivity of microalgal species. It is well established that *N. gaditana* and *P. tricornutum* are the most promising microalgal strains for lipid production and that they have higher growth rates than dinoflagellates (38). The specific growth rates in the same autotrophic cultures of *N. gaditana*, *P. tricornutum*, and *P. donghaiense* were not significantly different (Table 1), but *N. gaditana* and *P. tricornutum* had a longer exponential growth stage, and their cell densities reached 3.01 × 10^7^ and 1.07 × 10^7^ cells/ml during the stationary phase in the enclosed system (Fig. 1A to C). The cell density of *P. donghaiense* reached a concentration of 3.9 × 10^5^ cells/ml in a similar enclosed system, which was 77-fold lower than that of *N. gaditana* and 27-fold lower than that of *P. tricornutum* in a similar production system. However, the low cell density of *P. donghaiense* is compensated for by its large size and high biovolume and lipid productivity. *P. donghaiense* is large, and its biovolume is different from those of *N. gaditana* and *P. tricornutum*. Therefore, the biomass concentration of *P. donghaiense* (790.40 ± 23.78 mg liter^−1^) was 3.7-fold higher than that of *N. gaditana* (212.39 ± 11.87 mg liter^−1^) and 2.1-fold higher than that of *P. tricornutum* (369.11 ± 18.22 mg liter^−1^) (Table 1). This characteristic could be useful in terms of lipid storage. The lipid productivity of *P donghaiense* (95.47 ± 0.99 mg liter^−1^ day^−1^) is much higher than those of *N. gaditana* (23.81 ± 4.38 mg liter^−1^ day^−1^), *P. tricornutum* (39.06 ± 0.18 mg liter^−1^ day^−1^) (Table 1), and *Chlorella* (7.96 to 44.7 mg liter^−1^ day^−1^) (19, 39) and is similar to that of *Nannochloropsis* (37.6 to 158.76 mg liter^−1^ day^−1^) (12, 39). Although there were differences in cell abundances among species, the balance between cell abundance, biovolume, and lipid content led to final lipid production values in *P. donghaiense* that were much higher than those of *N. gaditana* and *P. tricornutum.* Hence, *P. donghaiense* is a promising oleaginous species for lipid production. Lipid productivity among the other dinoflagellate species was not significantly different, with the exception of that in A. carterae, which achieved higher lipid productivity at 10.92 ± 1.81 mg liter^−1^ day^−1^. *S. trochoidea*, which had a higher lipid content (27.36% ± 1.76%) than A. carterae (22.64% ± 0.75%), attained a low lipid productivity of only 2.13 ± 0.33 mg liter^−1^ day^−1^ because of its noncompetitive biomass productivity (Table 1). *P. donghaiense* was the best species for lipid accumulation in this study.

**TABLE 1:** Biomass productivity, lipid productivity and lipid content of 8 microalgal strains^*a*^

Algal species	Specific growth rate (day^−1^)	Generation time (days)	Biomass concn (mg liter^−1^)	Lipid content (% dry wt)	Lipid productivity (mg liter^−1^·day^−1^)
*Nannochloropsis gaditana*	0.28 ± 0.01	2.43 ± 0.11	212.39 ± 11.87	41.39 ± 3.95	23.81 ± 4.38
*Phaeodactylum tricornutum*	0.31 ± 0.01	2.21 ± 0.07	369.11 ± 18.22	33.58 ± 3.07	39.06 ± 0.18
*Alexandrium minutum*	0.16 ± 0.01	4.17 ± 0.27	53.42 ± 4.14	23.90 ± 1.48	2.17 ± 0.20
Amphidinium carterae	0.23 ± 0.01	2.96 ± 0.14	203.92 ± 13.42	22.64 ± 0.75	10.92 ± 1.81
*Scrippsiella trochoidea*	0.15 ± 0.01	4.52 ± 0.29	50.64 ± 2.51	27.36 ± 1.76	2.13 ± 0.33
*Prorocentrum micans*	0.12 ± 0.01	5.67 ± 0.78	140.32 ± 11.86	20.75 ± 1.66	3.23 ± 0.19
*Prorocentrum minimum*	0.18 ± 0.00	3.73 ± 0.14	109.04 ± 2.30	18.02 ± 0.10	4.47 ± 0.74
*Prorocentrum donghaiense*	0.24 ± 0.00	2.90 ± 0.03	790.40 ± 23.78	49.32 ± 1.99	95.47 ± 0.99

a Values are the means ±SDs (*n* = 5).

### Fatty acid compositional properties of 8 microalgal species.

**FIG 2:**
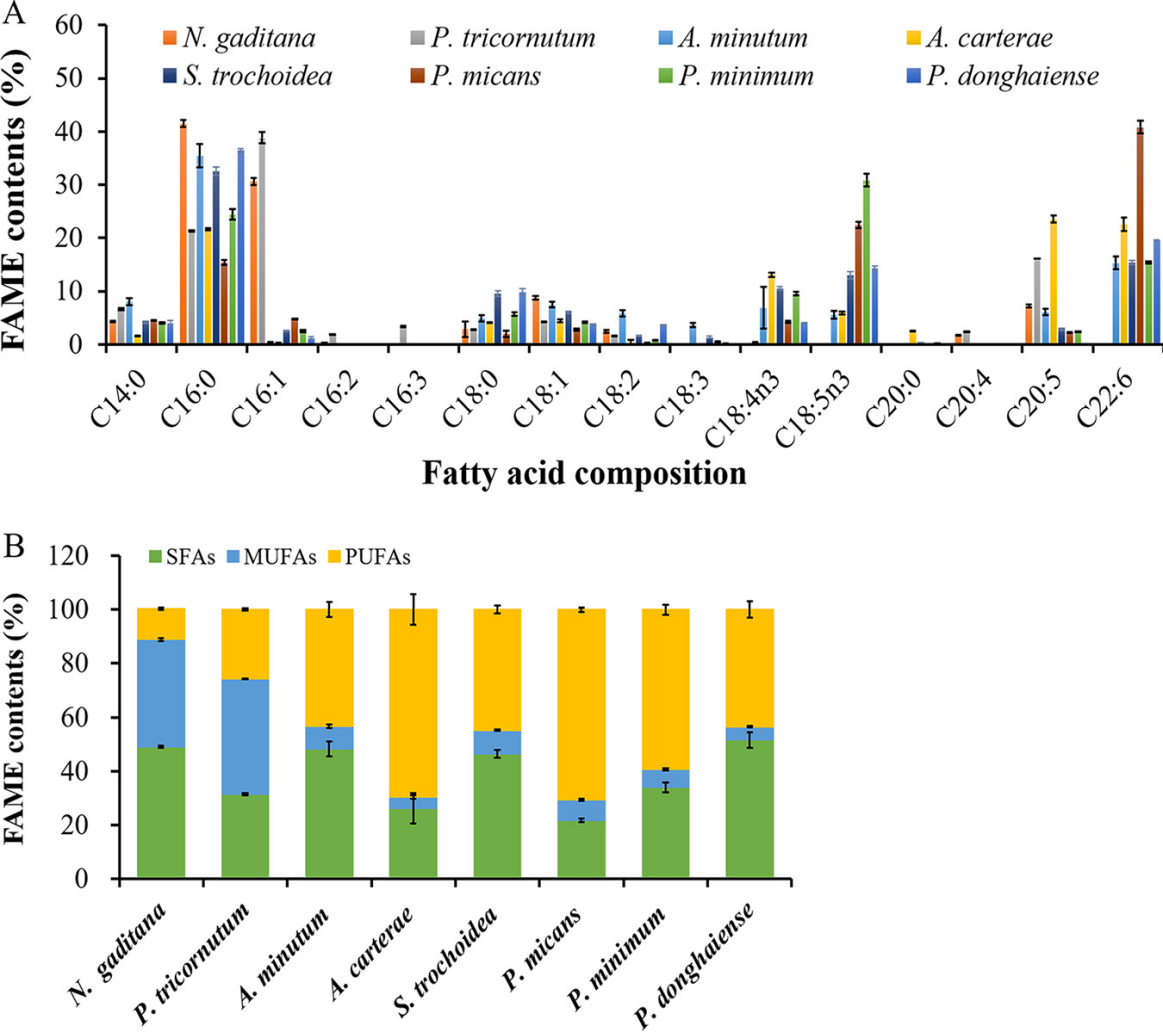
Comparison of the fatty acid composition of 8 microalgal strains. (A) Fatty acid compositional profiles. (B) The percentages of SFAs, MUFAs, and PUFAs in the total fatty acids. SFAs: C_14:0_, C_16:0_, C_18:0_, C_20:0_, and C_22:0_; MUFAs: C_16:1_, C_17:1_, C_18:1_, and C_22:1_; PUFAs: C_16:2_, C_16:3_, C_18:2_, C_18:3_, C_18:4_, C_18:5_, C_20:2_, C_20:4_, C_20:5_, and C_22:6_.

The fatty acid composition analysis indicated that the proportions of saturated, monounsaturated, and polyunsaturated compounds produced by the 8 algal species varied significantly. As shown in Fig. 2, *N. gaditana* had high proportions of saturated fatty acids (SFAs) and monounsaturated fatty acids (MUFAs), and the ratios of C_16:0_, C_16:1_, and C_18:1_ in *N. gaditana* accounted for 41.5% ± 0.64%, 30.66% ± 0.67%, and 8.74 ± 0.32% of the total lipids, respectively. In addition to high contents of C_16:0_ (21.35% ± 0.15%) and C_16:1_ (38.84% ± 1.07%), *P. tricornutum* contained a high proportion of the polyunsaturated fatty acid (PUFA) C_20:5_ (eicosapentaenoic acid [EPA]), which increased the degree of unsaturation (DU) of the lipids. The fatty acid composition of dinophyta was characterized by a high content of PUFAs. In addition to C_16:0_, all 6 dinoflagellate species in this study contained high contents of PUFAs, ranging from 43.22% ± 23.75% to 70.49% ± 0.77%. The two predominant PUFAs were C_22:6_ (docosahexaenoic acid [DHA]) and an unusual C_18:5n3_ (octadecapentaenoic acid), which are the main biomarkers of photosynthetic dinoflagellates (40). PUFAs are known mostly for their nutritional importance to the development and function of the brain and visual systems. DHA plays essential roles in regulating neural development and exerts beneficial effects against cardiovascular, neurological, and proliferative diseases (41). Current natural sources of DHA are limited or of unsatisfactory quality for human nutrition, with most DHA obtained from fish oil, which can be contaminated with heavy metals, antibiotics, and pesticides. Such contamination could be addressed by refining fish oil, but the refinement process would result in even higher prices due to the greater amount of raw material needed for processing and the higher operating costs. Thus, providing the recommended PUFA levels to a growing world population calls for additional sustainable supplies of oils of sufficient quality for human consumption. The accumulation of oil that is highly enriched in PUFAs is rare in microalgae (42). Among the studied oleaginous microalgal species, the genus *Nannochloropsis* and *P. tricornutum* contain high concentrations of EPA (25), and only Schizochytrium limacinum has been reported to have a high content of DHA (5). It has been reported that a genetically engineered strain of *P. tricornutum* produces maximum yields of DHA and EPA of 36.5% and 23.6% of the total fatty acids, respectively, indicating the suitability of this species for the commercial production of EPA and DHA (43). Hence, the high contents of PUFAs, especially DHA, make photoautotrophic dinoflagellates, especially *P. micans*, *P. donghaiense*, and A. carterae, potentially good sources of nutrients for humans. A. carterae, which was enriched in both EPA (23.52% ± 0.65%) and DHA (22.58% ± 1.28%), is suitable for the commercial production of EPA and DHA. *P. donghaiense*, with its high lipid productivity and high DHA proportion, is suitable for DHA production. Although *P. micans* had a higher proportion of DHA (40.83% ± 1.21% of its total lipids), it is not a good source for commercial DHA production because of its low lipid content and productivity.

A decrease in fatty acid DU alters the fluidity of biological membranes and impacts the activities of various integral membrane proteins but is conducive to biodiesel production. To evaluate the potential of 8 microalgal species for biodiesel production, the key parameters affecting biodiesel quality were evaluated on the basis of the fatty acid methyl ester (FAME) composition. Cetane number (CN) is a prime indicator of biodiesel quality because it is related to nitrous oxide emissions, engine performance, and diesel fuel combustion efficiency (44). A high CN indicates better ignition properties and engine performance. Therefore, the biodiesel specification standard ASTM D6751 recommends a CN of at least 47 for diesel used as an engine fuel (45). Iodine value (IV) is related to the DU of fatty acids and the oxidative stability and cold flow properties of diesel. The European standard EN 14214 recommends a maximum IV of 120 g I_2_ 100 g^−1^ (46). As shown in Table 2, only *N. gaditana* was the best raw material for biodiesel production because of its higher CN and lower IV. Although *P. tricornutum* produced lipids with high proportions of SFAs and MUFAs among total lipids, the high content of PUFAs increased the DU of lipids; therefore, the CN and IV of the total lipids did not meet the biodiesel specification standards. Because they had IVs higher than 120 g I_2_ 100 g^−1^ and CNs lower than 47, none of the 6 dinoflagellate species were suitable for biodiesel production.

**TABLE 2:** Biodiesel properties of lipids of 8 microalgal strains^*a*^

Strain	DU (%)	IV (g I_2_/100 g)	SV	CN
Green algae				
*N. gaditana*	61.92 ± 0.35	74.59 ± 0.17	203.58 ± 0.09	56.32 ± 0.02
Diatom				
*P. tricornutum*	94.76 ± 0.03	130.47 ± 0.33	202.53 ± 0.05	43.89 ± 0.06
Dinoflagellate				
*A. minutum*	93.01 ± 5.41	163.72 ± 11.18	195.44 ± 0.88	37.38 ± 2.39
A. carterae	135.47 ± 0.35	272.45 ± 1.23	185.5 ± 0.25	14.42 ± 0.23
*S. trochoidea*	98.35 ± 3.44	189.08 ± 7.3	194.07 ± 0.31	31.87 ± 1.59
*P. micans*	149.02 ± 2.29	314.26 ± 4.34	185.37 ± 0.27	5.03 ± 0.93
*P. minimum*	122.71 ± 0.8	250.53 ± 2.16	193.69 ± 0.08	18.1 ± 0.47
*P. donghaiense*	89.84 ± 0.71	178.54 ± 1.63	193.45 ± 0.14	34.33 ± 0.34

a DU, degree of unsaturation {(monounsaturated fatty acids [wt%]) + 2(polyunsaturated fatty acids [wt%])}; IV, iodine value; SV, saponification value; CN, cetane number.

### Effect of nutrient deficiency on the biomass, lipid content, and fatty acid composition of *P. donghaiense*.

**FIG 3:**
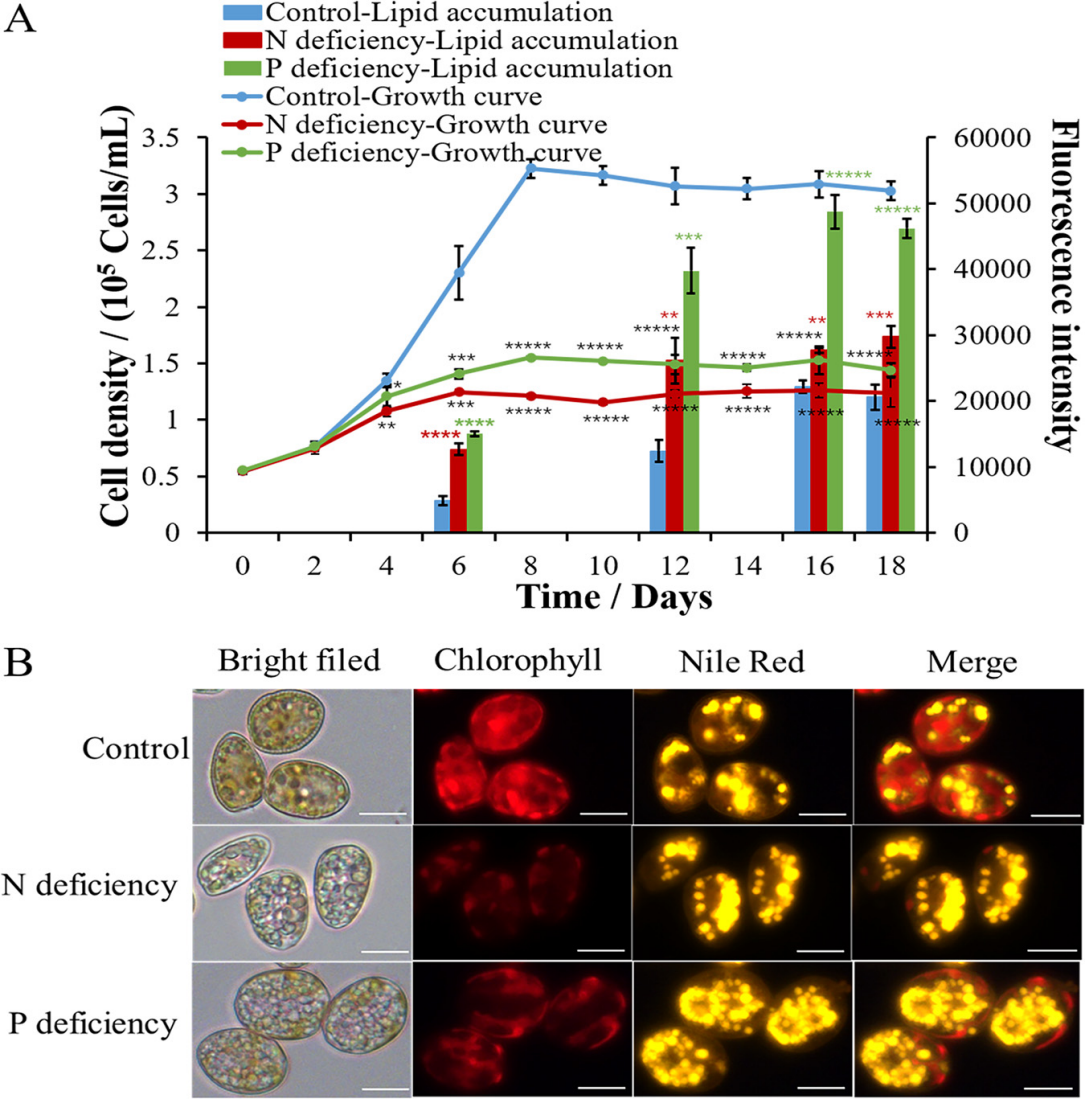
Growth, lipid accumulation, and fatty acid compositions of *P. donghaiense* under N- and P-deficient conditions. (A) Growth and lipid accumulation of *P. donghaiense* under different conditions. (B) Photographs of algal cells under different conditions. Lipid droplets were stained with Nile red and observed with a fluorescence microscope. The values are the means ±SDs (*n* = 3). *, *P* < 0.05; **, *P* < 0.01; ***, *P* < 0.001; ****, *P* < 0.0001; *****, *P* < 0.00001 versus the control.

**FIG 4:**
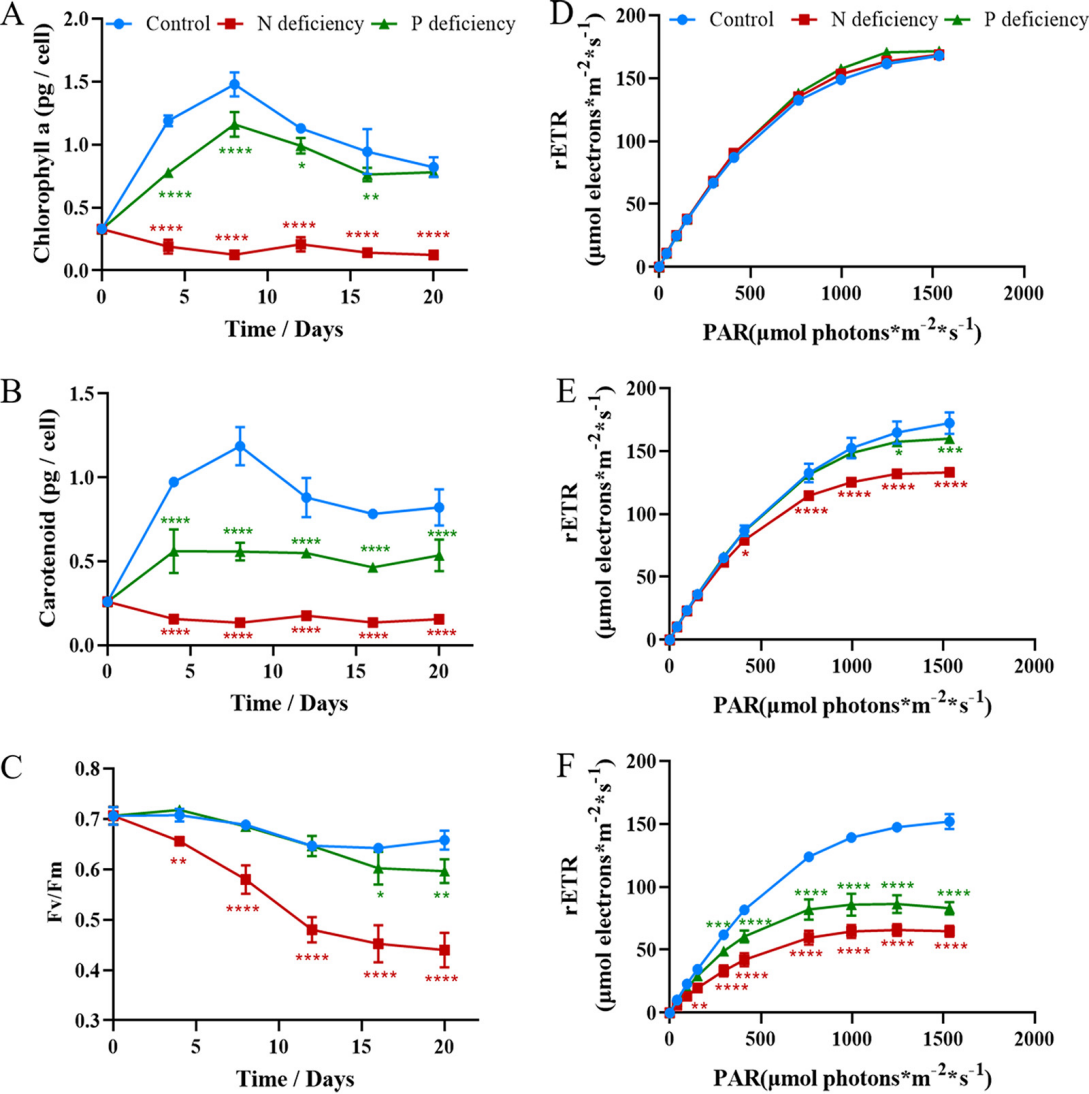
Photosynthetic response of *P. donghaiense* to N- and P-deficient conditions. Chlorophyll *a* contents (A), carotenoid contents (B), Fv/Fm (C), rETR at day 0 (D), rETR at day 4 (E), and rETR at day 12 (F). The values are the means ±SDs (*n* = 3). *, *P* < 0.05; **, *P* < 0.01; ***, *P* < 0.001; ****, *P* < 0.0001 versus the control.

**FIG 5:**
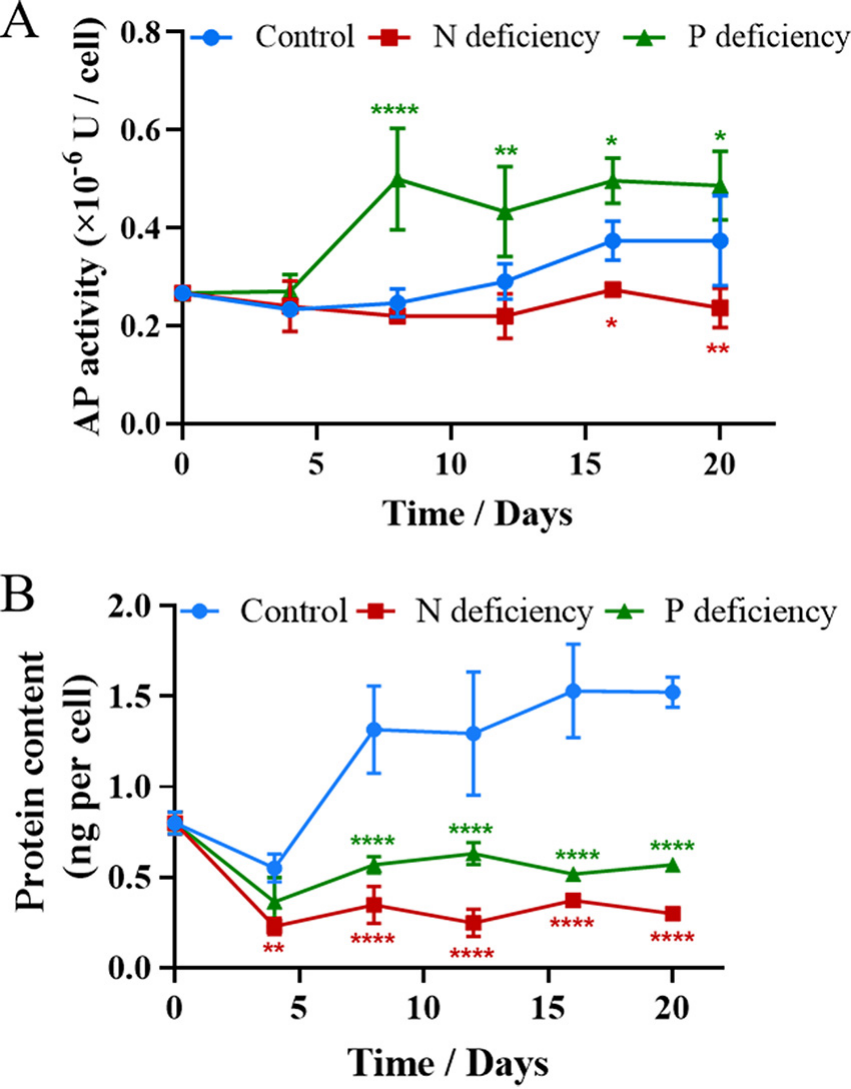
Alkaline phosphatase activities (A) and protein contents (B) of *P. donghaiense* under N- and P-deficient conditions. AP, alkaline phosphatase. The values are the means ±SDs (*n* = 3). *, *P* < 0.05; **, *P* < 0.01; ****, *P* < 0.0001 versus the control.

Nutrient limitation is a widely utilized strategy to induce lipid accumulation. N (47–49) and P (10) are commonly reported to be the limiting nutrients that trigger lipid accumulation in microalgae. To evaluate the maximum potential for lipid production by *P. donghaiense*, algal cells were cultivated in N- or P-deficient medium. As shown in Fig. 3A, both N starvation and P starvation remarkably enhanced lipid accumulation in *P. donghaiense*. The Nile red fluorescence intensities of the N-deficient and P-deficient cultures reached maximum values on day 12, which were 2-fold and 3-fold that in the control. Intracellular lipid droplets were visualized by fluorescence microscopy. Compared to that in the control, the numbers of lipid droplets significantly increased in N-deficient cultures and in P-deficient cultures (Fig. 3B). Although nutrient limitation induced lipid accumulation in *P. donghaiense*, it significantly decreased the microalgal biomass of *P. donghaiense*. As shown in Fig. 3A, N deficiency and P deficiency remarkably inhibited the growth of *P. donghaiense* and shortened the exponential phase, which impacted the final cell density. The number of *P. donghaiense* cells under P deficiency was 2-fold lower than that in the control. Compared with P deficiency, N deficiency was a greater stressor and completely stopped the growth of *P. donghaiense.* The number of *P. donghaiense* cells under N deficiency was only 37.5% of the number of cells in the control. Moreover, the algal cells under P deficiency exhibited larger cell sizes and stronger chlorophyll (Chl) fluorescence than the algal cells under N deficiency (Fig. 3B). Although the stress due to N deficiency was more severe in *P. donghaiense* than that due to P deficiency, the latter induced greater lipid production. This result goes against generally accepted trends. Typically, P starvation exerts less pronounced effects on neutral lipid accumulation than N starvation (50). Based on the results shown in Fig. 3B, we thought that both N and P deficiency could inhibit cell proliferation and cause a metabolic shift to lipid accumulation; however, algal cells under P deficiency had stronger Chl fluorescence, indicating a higher photosynthetic capacity to produce more carbon hydrate for lipid accumulation than algal cells under N deficiency. To confirm our inference, photosynthetic pigments and capacity were detected. As shown in Fig. 4, the contents of Chl *a* and carotenoids in algal cells under N and P deficiency significantly decreased, but the Chl *a* and carotenoid contents in the P-deficient culture were significantly higher than those in N-deficient culture. Accordingly, the maximum quantum yield of photosystem (PS) II (Fv/Fm) and the relative electron transport rate (rETR) in the P-deficient culture were markedly higher than those in the N-deficient culture. These results indicated that the photosynthetic capacity of algal cells under P stress is much stronger than that of algal cells under N stress and that algal cells can produce more carbon hydrate to accumulate lipids. The stronger photosynthetic capacity is probably due to the enhanced activity of alkaline phosphatase of *P. donghaiense* under P deficiency. Ou et al. reported that *P. donghaiense* suffered P stress and expressed abundant alkaline phosphatase, which might help it efficiently utilize organic phosphorus substrates and outcompete other concurrent species to outburst in the P-deficient East China Sea (51). We also found that alkaline phosphatase activities markedly increased after algal cells were cultured in P deficiency for 4 days and peaked at day 8, which was 2 times that of the control. Then, the activity of alkaline phosphatase always remained higher than that in control cells (Fig. 5A). The results suggested that *P. donghaiense* could partly alleviate the adverse effects on the photosynthetic system exerted by P deficiency by increasing alkaline phosphatase to produce more carbohydrates for lipogenesis. The activity of alkaline phosphatase in the N-deficient culture was markedly lower than that in control cells (Fig. 5A), which was probably due to the inhibition of protein synthesis and promotion of protein catabolism under N deficiency. As shown in Fig. 5B, the protein content of algal cells cultured in N deficiency was far lower than those of the control group and P-deficient culture. Yao et al. reported that branched-chain amino acid catabolism fueled acetyl coenzyme A (acetyl-CoA) production for tricarboxylic acid (TCA) metabolism and lipogenesis (52). Thus, we think that lipid accumulation under N deficiency is due to protein catabolism and amino acid recycling. Chungjatupornchai et al. reported that overexpression of plastidial lysophosphatidic acid acyltransferase considerably increased triacylglycerol (TAG) content and productivity in Neochloris oleoabundans (53). We thus think that lipid accumulation under P deficiency is due to phospholipid catabolism and alkaline phosphatase activity increase. On the basis of the above-described results, we concluded that lipid accumulation in *P. donghaiense* was regulated in different ways under N and P deficiency.

To evaluate the biomass and lipid productivity, we determined the total lipid contents of *P. donghaiense* grown under different culture conditions. As shown in Table 3, the biomass concentration and lipid productivity of *P. donghaiense* cultured in normal medium were 700.67 ± 9.72 mg liter^−1^ and 101.81 mg liter^−1^ day^−1^, respectively. The total lipid content reached 54.7% ± 0.06% of the dry weight. Although the biomass concentration of algal cells grown under N- and P-deficient conditions decreased significantly to 260.71 ± 10.81 and 341.78 ± 1.08 mg liter^−1^, respectively, their lipid contents significantly increased, with their total lipid contents reaching 74.96% ± 1.11% and 79.37% ± 1.84% of dry weight, respectively. These results indicated that N deficiency and P deficiency induced lipid accumulation. N and P deficiency along with the availability of carbon sources divert the cellular carbon flux from protein to fatty acid synthesis, which increases lipid content in algae; however, such deficiencies eventually lead to the inhibition of cell growth and cell division (54). Although nutrient stress inhibited cell growth, the lipid productivity (50.95 ± 3.72 mg liter^−1^ day^−1^) of *P. donghaiense* under P-deficient conditions was still much higher than that of *N. gaditana* and *P. tricornutum* under normal conditions (Table 1). The lipid productivity (27.6 ± 4.98 mg liter^−1^ day^−1^) of *P. donghaiense* under N-deficient conditions was similar to that of *N. gaditana* under normal conditions.

**TABLE 3:** Biomass productivity, lipid productivity, and lipid contents of *P. donghaiense* cultured under different nutrient conditions

Photoautotrophic condition	Specific growth rate (μ day^−1^)	Generation time (days)	Biomass concn (mg liter^−1^)	Lipid content (% dry wt)	Lipid productivity (mg liter^−1^·day^−1^)
Control	0.25 ± 0.00	2.77 ± 0.01	700.67 ± 9.72	54.7 ± 0.06	92.5 ± 5.5
N deficiency	0.16 ± 0.00	4.41 ± 0.01	260.71 ± 10.81	74.96 ± 1.11	27.6 ± 4.98
P deficiency	0.18 ± 0.00	3.82 ± 0.07	341.78 ± 1.08	79.37 ± 1.84	50.95 ± 3.72

### Effect of nutrient deficiency on the fatty acid composition of *P. donghaiense*.

**FIG 6:**
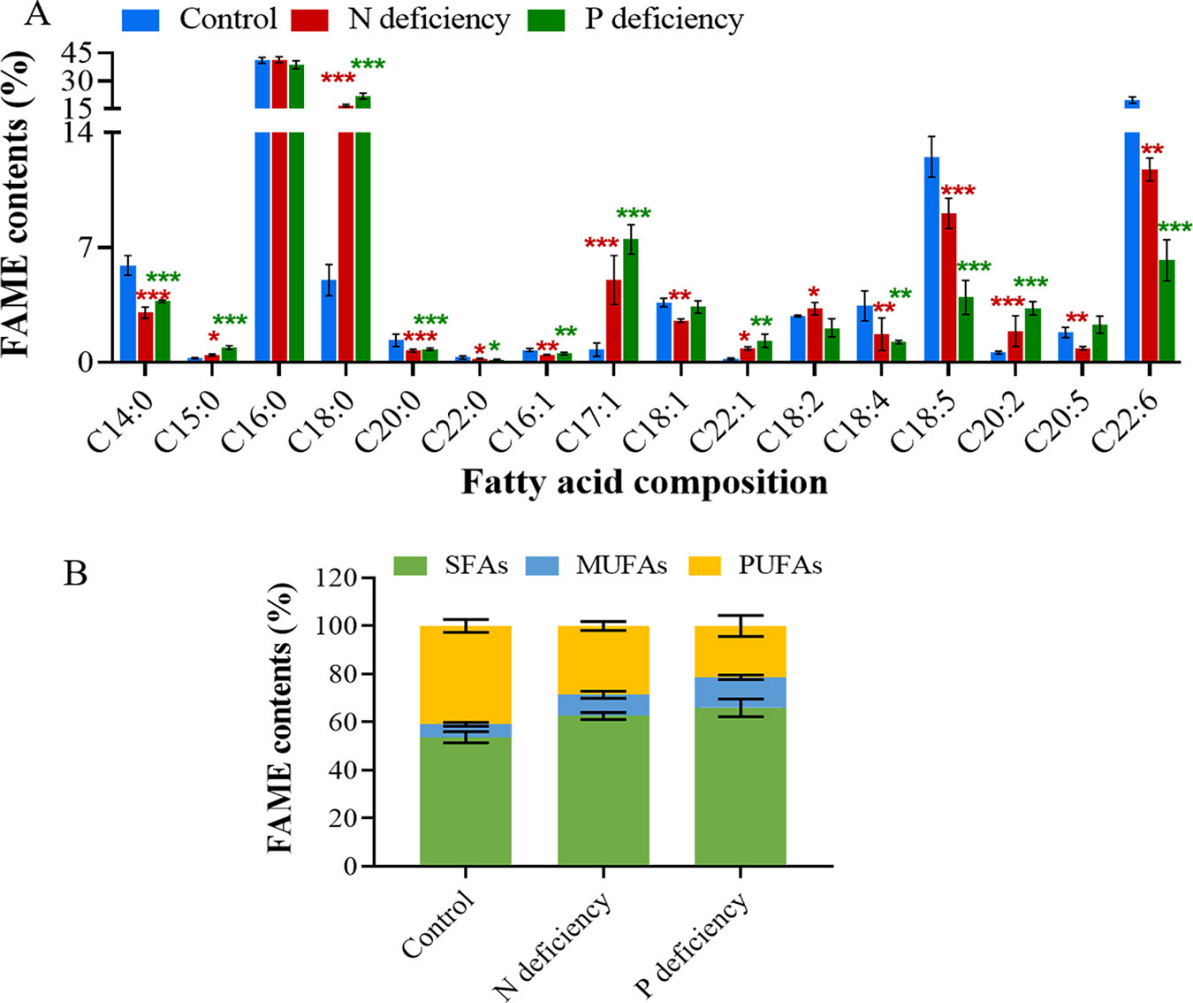
Fatty acid compositions of *P. donghaiense* under different culture conditions. (A) Fatty acid compositional profiles. (B) Percentages of SFAs, MUFAs, and PUFAs among the total fatty acids. SFAs: C_14:0_, C_15:0_, C_16:0_, C_18:0_, C_20:0_, and C_22:0_; MUFAs: C_16:1_, C_17:1_, C_18:1_, and C_22:1_; PUFAs: C_18:2_, C_18:4_, C_18:5_, C_20:2_, C_20:5_, and C_22:6_. The values are the means ±SDs (*n* = 3). *, *P* < 0.05; **, *P* < 0.01; ***, *P* < 0.001 versus the control.

The lipid profiles of *P. donghaiense* cultured under different nutrient conditions were analyzed by gas chromatography-mass spectrometry (GC-MS). As shown in Fig. 6, *P*. *donghaiense* grown in normal cultures accumulated high proportions of SFAs and PUFAs. The total contents of SFAs and PUFAs reached 53.74% ± 1.89% and 40.86% ± 2.12% of the total fatty acids, respectively, while that of MUFAs reached only 5.33% ± 0.65%. The most abundant SFA was palmitic acid (C_16:0_), which accounted for 40% of the total fatty acids, followed by myristic acid (C_14:0_) and stearic acid (C_18:0_), which accounted for 5.9% and 5%, respectively. The two predominant PUFAs were C_22:6n3_ (19.7% ± 1.34%) and C_18:5n3_ (12.51% ± 1.01%).

The fatty acid composition of microalgae can be affected by various culture conditions, such as different nutrient conditions and environmental factors. PUFAs play a prominent role in protecting membranes from rigidification or solidification and support effective electron transport chains in chloroplasts. Maintenance of a high unsaturation level is imperative for the survival of microalgae under adverse conditions, such as low-temperature, high-light, osmotic, oxidative, and nutrient stresses (50). Many studies have reported that nutrient stress alters the fatty acid composition of algal cells and causes high fatty acid unsaturation levels. For example, N deficiency increased the proportions of C_16:1_, C_18:1_, and C_20:5_ in *Tribonema* sp. (55). P deprivation increased the contents of the PUFAs C_18:2_, C_20:3_, C_20:4_, and C_20:5_ in Porphyridium cruentum (56). Interestingly, our results showed the opposite change in the fatty acid composition of *P. donghaiense* under nutrient stress. As shown in Fig. 6, both N starvation and P starvation increased the SFA and MUFA contents and decreased the PUFA levels in *P. donghaiense*. Highly significant differences were observed among the normal cultures, N-deficient cultures, and P-deficient cultures in terms of their contents of SFAs, MUFAs, and three PUFAs (Fig. 6). The contents of a C_18_ SFA (C_18:0_, stearic acid) and a C_17_ MUFA (C_17:1_, margaric acid) increased by 3.4-fold and 5.6-fold, respectively, in the N-deficient cultures and by 4.5-fold and 7.3-fold, respectively, in the P-deficient cultures; the contents of PUFAs (C_18:4n3_, C_18:5n3_, and C_22:6_) decreased significantly (*P* < 0.01) in both N-deficient cultures and P-deficient cultures. Three PUFAs (C_18:4n3_, C_18:5n3_, and C_22:6_) decreased by 50.1%, 27.5%, and 40.4%, respectively, in the N-deficient cultures and by 65%, 67.8%, and 69.3%, respectively, in the P-deficient cultures. Accordingly, the DU decreased significantly by 24.4% in the N-deficient cultures and by 40.8% in the P-deficient cultures (Table 4). These results indicated that stress due to N deficiency or P deficiency caused PUFAs to decrease and SFAs and MUFAs to increase in *P. donghaiense* and ultimately resulted in a decrease in the unsaturation level of the total lipids. The green oleaginous microalga Lobosphaera incisa was reported to accumulate lipids enriched in long-chain PUFAs under N deprivation and accumulate lipids enriched in MUFAs under P deprivation (50). Our results showed that the production of SFAs and MUFAs prevailed in *P. donghaiense* under both N deprivation and P deprivation. The results indicated that microalgae have acquired different strategies, including carbon reallocation and lipid remodeling, to cope with a lack of essential nutrients, such N and P.

**TABLE 4:** Effect of different treatments on the biodiesel properties of lipids of *P. donghaiense*^*a*^

Treatment	DU (%)	IV (g I_2_/100 g)	SV	CN
Control	87.71 ± 4.75	175.04 ± 9.93	194.3 ± 0.2	35 ± 2.2
N deficiency	65.93 ± 2.42	118.11 ± 6.26	195.24 ± 0.37	47.67 ± 1.39
P deficiency	51.59 ± 4.8	97.32 ± 6.59	196.11 ± 0.32	56.29 ± 2.53
1% Y42 supernatant	82.66 ± 4.67	163.12 ± 7.15	194.97 ± 1.57	38.22 ± 2.19
3% Y42 supernatant	35.3 ± 6.42	47.53 ± 3.83	198.61 ± 3.29	63.47 ± 3.01
5% Y42 supernatant	36.22 ± 7.47	31.69 ± 4.52	205.19 ± 4.31	66.38 ± 3.86

a DU, degree of unsaturation {(monounsaturated fatty acids [wt%]) + 2(polyunsaturated fatty acids [wt%])}; IV, iodine value; SV, saponification value; CN, cetane number.

### Biodiesel properties of *P. donghaiense* under different nutrient conditions.

In general, lipids with a high proportion of SFAs and MUFAs resulted in better biodiesel quality (16). To evaluate the potential of *P. donghaiense* cultured under N- and P-deficient conditions for biodiesel production, the key parameters affecting biodiesel quality were evaluated on the basis of the FAME composition of the lipids. As shown in Table 4, although the lipids of *P. donghaiense* had a CN lower than 47 and an IV higher than 120 g I_2_ 100 g^−1^ during the acclimation period, the CN and IV were altered by N deficiency and P deficiency. The total lipids of *P. donghaiense* in N-deficient and P-deficient cultures had CNs that all exceeded 47 and IVs that were all below 120 g I_2_ 100 g^−1^. These values are in accordance with the ASTM D6751 and EN 14214 standards; thus, these cultures are favorable for biodiesel production. Hence, using N-deficient or P-deficient cultures could improve the lipid quality of *P. donghaiense* enough to reach biodiesel specification standards. Wu and Miao reported that better quality biodiesel could be obtained from *Chlorella pyrenoidosa* (Auxenochlorella pyrenoidosa) in the absence of nitrate from Scenedesmus obliquus at low nitrate concentrations. Thus, they thought that growing microalgae in the presence of nitrogen may limit the resultant biodiesel quality (16). Our results also demonstrated that N deficiency could improve the biodiesel quality of *P. donghaiense* for biodiesel. However, the best biodiesel quality was obtained from *P. donghaiense* under P deficiency. Due to its higher lipid production than that for *N. gaditana* and *P. tricornutum* (Tables 1 and 3), *P. donghaiense* cultured under P-deficient conditions is a good microalgal source for biodiesel production.

### Effects of the supernatant of the algal-lytic bacterium *Paracoccus* sp. Y42 on *P. donghaiense* cell lysis, lipid content, and fatty acid composition.

**FIG 7:**
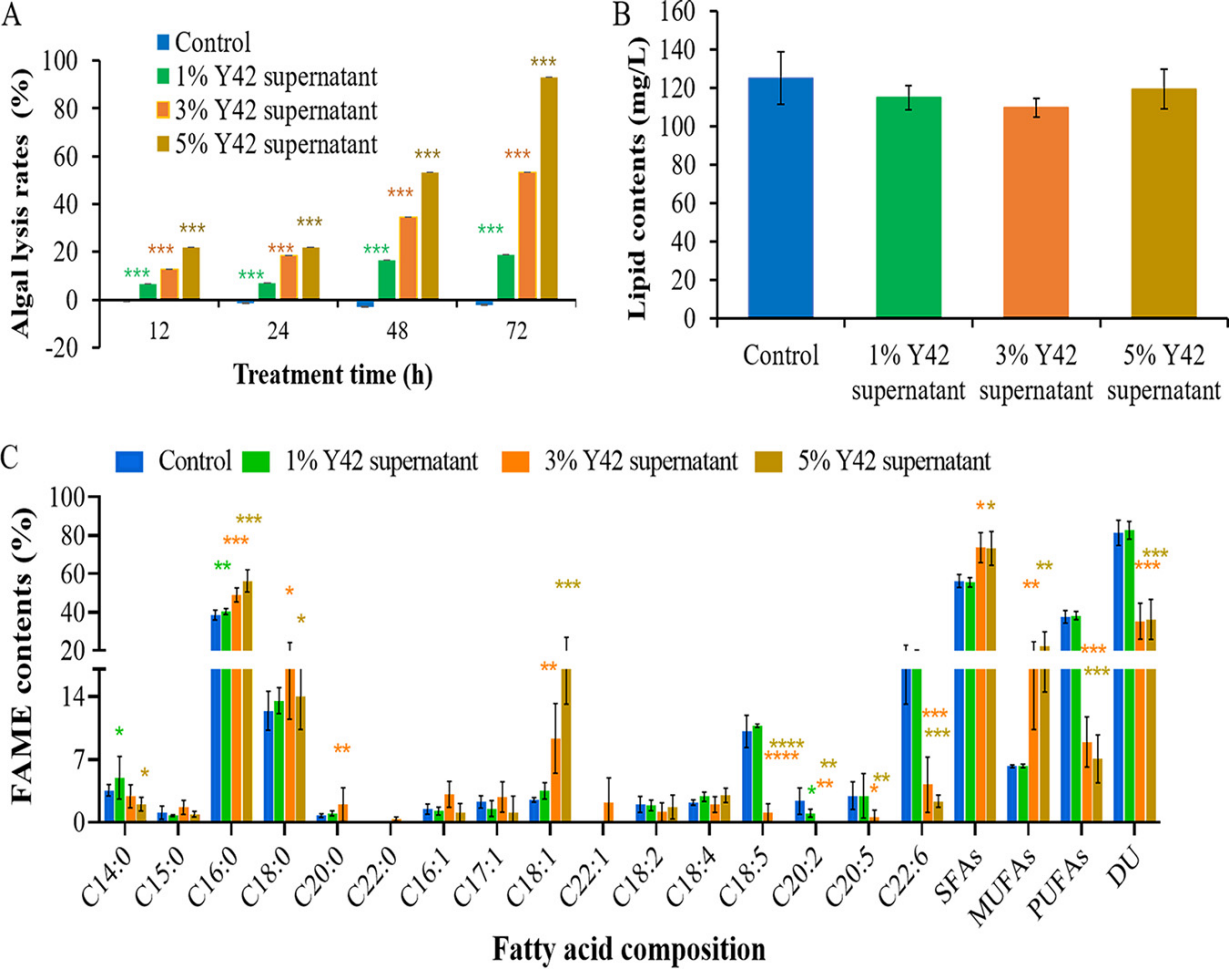
Effects of the algal-lytic bacterium *Paracoccus* sp. Y42 on the lipid content and fatty acid composition of *P. donghaiense*. (A) Algal-lytic activities of different concentrations of the Y42 supernatant. (B) Lipid contents of algal cells treated with different concentrations of the Y42 supernatant for 72 h. (C) Fatty acid compositions of algal cells treated with different concentrations of the Y42 supernatant for 72 h. Values are the means ± SDs (*n* = 3). *, *P* < 0.05; **, *P* < 0.01; ***, *P* < 0.001; ****, *P* < 0.0001 versus the control.

**FIG 8:**
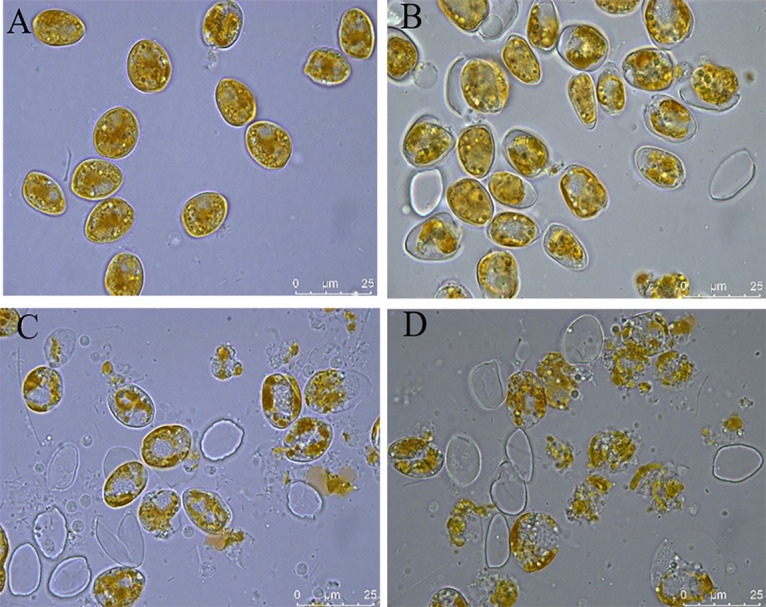
Efficiency of the Y42 supernatant to lyse *P. donghaiense*. (A) *P. donghaiense* culture. (B) *P. donghaiense* lysed by 1% Y42 supernatant. (C) *P. donghaiense* lysed by 3% Y42 supernatant. (D) *P. donghaiense* lysed by 5% Y42 supernatant.

To evaluate the potential of the algicidal bacterium *Paracoccus* sp. Y42 for the downstream extraction process and lipid production, the algal-lytic efficiency and effects on the lipid content and fatty acid composition of *P. donghaiense* after treatment with the Y42 supernatant for 72 h were examined. As shown in Fig. 7A, the algicidal rates increased with increasing treatment time and with enhanced concentrations of the Y42 supernatant. Nearly 95% of algal cells were lysed after treatment with 5% Y42 supernatant for 72 h (Fig. 8), indicating that this treatment of the algal culture can directly extract lipids without mechanical breaking. As shown in Fig. 7B, the lipid contents of the 5% treatment group were not significantly different (*P* > 0.05) from those of the control, 1%, and 3% treatment groups, which were disrupted by sonicating 100 times before extracting lipids. This result indicated that the algicidal bacterium Y42 can facilitate the downstream extraction process and decrease the associated energy consumption.

To investigate the effect of the algal-lytic bacterium on lipid profiles, the fatty acid composition of *P. donghaiense* was analyzed. As illustrated in Fig. 7C, highly significant differences in the contents of SFAs, MUFAs, and PUFAs were observed between the control and Y42-treated cultures. The algal cells in the control and 1% treatment groups accumulated high concentrations of SFAs and PUFAs. The total SFA and PUFA contents reached 56.26% ± 3.41% and 37.51% ± 3.25% of the total fatty acid contents, respectively. The DUs were higher than 80 (Table 4). The PUFA contents of *P. donghaiense* in the 3% and 5% Y42 treatment groups significantly decreased by 4-fold, and the contents of SFAs and MUFAs increased by 50% and 3-fold, respectively (Fig. 6C and Table 4). Accordingly, their DUs decreased markedly to 35 to 36. Thus, the total lipids of *P. donghaiense* in the 3% and 5% Y42 supernatant treatments had CNs that all exceeded 47 and IVs that were all below 120 g I_2_ 100 g^−1^ (Table 4). These values are the ASTM D6751 and EN 14214 standards, indicating that *P. donghaiense* is appropriate for biodiesel production. These results suggest that 5% Y42 supernatant not only lysed more than 95% of algal cells to simplify the lipid extraction process but also induced oxidative stress in *P. donghaiense* to decrease its PUFA content and increase its SFA and MUFA contents. These effects ultimately resulted in a decrease in the unsaturation level of the total lipids and benefited biodiesel production.

### Conclusions.

This study evaluated the lipid productivity of 8 microalgal species and demonstrated the great potential of *P. donghaiense* as a source of DHA for human nutritional products and as a source of biofuel because of its ability to generate large amounts of biomass and accumulate 50% to 80% of its dry weight as storage oil. The properties of the lipids produced by *P. donghaiense* grown in N- or P-deficient medium match biodiesel specification standards. *Paracoccus* sp. Y42 not only lysed algal cells, which facilitated the downstream lipid extraction process and saved energy, but also improved the suitability of the lipid profile of *P. donghaiense* for biodiesel production without affecting the lipid contents of *P. donghaiense*.

## MATERIALS AND METHODS

### Strain cultures.

*Alexandrium minutum*, Amphidinium carterae, *Prorocentrum. donghaiense*, *P. micans*, *P. minimum*, *Scrippsiella trochoidea*, *Phaeodactylum tricornutum*, and *Nannochloropsis gaditana* were obtained from the Culture Collection Center of Marine Algae of the State Key Laboratory of Marine Environmental Science at Xiamen University, China. The algal culture was maintained in sterile f/2 medium prepared with natural seawater at 20 ± 1°C with illumination at a light intensity of approximately 50 μmol photons m^−2^ s^−1^ under a 12:12 h light-dark cycle. Nitrogen (N)-deficient medium was produced by omitting NaNO_3_ from the medium, and phosphorus (P)-deficient medium was produced by omitting NaH_2_PO_4_·H_2_O. Cell growth was determined by counting with a Countstar automated cell counter (Countstar IC1000; ALIT Life Science, Shanghai, China).

The algicidal bacterium *Paracoccus* sp. Y42 was cultured in liquid Zobell 2216E medium at 28°C with shaking at 150 rpm. The algal lysis assay was performed as described in our previous publication (57).

### Nile red fluorescence analysis.

Algal cells were harvested by centrifugation at 6,000 × *g* for 10 min and then washed twice with 0.01 M phosphate-buffered saline (PBS; pH 7.4). The cell pellet was resuspended in 1 ml of PBS (0.01 M, pH 7.4) and then incubated with 0.6 μg/μl Nile red for 10 min in the dark. Algal cells stained with Nile red were observed by a fluorescence microscope (Olympus BX41; Olympus, Tokyo, Japan) with excitation at 488 nm and emission at 550 nm. The fluorescence intensities of Nile red were measured with a flow cytometer (Fortessa; BD Biosciences, USA) using 488-nm excitation and a 550-nm bandpass filter. Approximately 1 × 10^5^ cells were injected and recorded. The results were analyzed using FlowJo software version 7.6 (FlowJo, LLC, USA).

### Determination of the growth rate, dry biomass, and lipid content.

The specific growth rates of 8 microalgal species were calculated according to the following formula:

$$\text{μ}=\left(\text{ln}N_{t}-\text{ln}N_{0}\right)/\left(t-t_{0}\right)$$

where μ (day^−1^) is the specific growth rate in the exponential growth phase, *N*_0_ is the cell density at the beginning of the exponential growth phase (*t*_0_), and *N_t_* is the cell density at the time (*t*) of the exponential phase.

Fresh algal cells were harvested through centrifugation. The dry weight of the algal biomass was determined after freeze-drying overnight. Biomass productivity was calculated as the dry biomass produced in the exponential growth phase. For the biomass productivity determination, algal cells were collected at the end of the exponential phase, and the following formula was applied (19): biomass productivity = dry weight × μ.

Lipids were extracted by solvent and determined gravimetrically. A 450-ml sample was extracted with a modified chloroform-methanol-water solvent system (58). Algal cultures in the stationary phase were used for lipid analysis. The cell pellets were freeze-dried and weighed to determine their dry biomass (W1). A total of 400 ml of algal culture was extracted 3 times with 100 ml of a chloroform-methanol (2:1) mixture. The lipid extracts were dried to a constant weight (W2) and dissolved in 1 ml of chloroform for the analysis of the fatty acid composition of the extracellular lipid extracts (13). Finally, 0.2 mg of a C_19:0_ solution was added as an internal standard. The lipid content was calculated as follows: lipid content (%) = W3/W1 × 100.

Lipid productivity was calculated according to the following equation: lipid productivity = biomass productivity × lipid content.

### Methyl esterification of fatty acids.

The lipid extracts were transferred into methanolysis tubes. After the evaporation of chloroform *in vacuo*, the lipid samples were methylated with 2 ml of 1% sulfuric acid (H_2_SO_4_) in methanol (vol/vol) at 80°C for 1 h. Then, 5 ml of ultrapure water was added to the solution to stop the methanolysis reaction, and fatty acid methyl esters (FAMEs) were extracted with 2 ml of *n*-hexane. The tube was vortexed and then incubated for at least 20 min at room temperature until phase separation. The hexane phase was directly analyzed by gas chromatography-mass spectrometry (GC-MS).

### Quantification of FAMEs by GC.

GC-MS analysis was performed on a Varian 1200 system (Varian Medical Systems, Palo Alto, CA, USA). One microliter of sample was injected into a Trace DCQ system containing a DB-5 column (30 m by 0.125 mm by 0.125 μm), with a split ratio of 1:20. The oven temperature was programmed from 150°C to 250°C at a rate of 3°C/min and finally held at 250°C for 2 min. The carrier gas was helium, with a flow rate of 0.18 ml/min. Each FAME was identified by comparison of its retention time with that of a standard. Each FAME was quantified by the surface peak method using the C_19_ surface peak for calibration with the following equation: measured quantity of FAMEs = area of FAME peak × measured quantity of C_19_/area of C_19_ peak.

### Evaluation of biodiesel properties.

Biodiesel properties, including the saponification value (SV), iodine value (IV), cetane number (CN), and degree of unsaturation (DU), were estimated. The percentages of monounsaturated fatty acids (MUFAs) and polyunsaturated fatty acids (PUFAs) were calculated from the FAME analysis.

The following calculations were used to estimate each parameter (59, 60):

DU=(MUFA [wt%]) + (2×PUFA [wt%])

$$\text{IV }=\Sigma (254\times \text{F}\times \text{D})/M_{\text{w}}$$

$$\text{SV}=\Sigma (560\times \text{F})/M_{\text{w}}$$

CN =(46.3 + 5,458/SV) − (0.225×IV)

where F is the percentage of each FAME, *M*_w_ is its molecular weight, and D is the number of double bonds in each FAME structure.

### Measurements of photosynthetic pigment contents.

Pigments were extracted with 90% alcohol after *P. donghaiense* was collected by centrifugation. The chlorophyll and carotenoid contents were determined by measuring the absorbance at 470 nm, 645 nm, and 665 nm in a 1-cm cuvette, and the chlorophyll *a* (Chl *a*) and carotenoid contents were estimated according to the following equations:

$$\text{Chl }a\left(\text{mg}/\text{liter}\right)\;=\text{ 12}.7\times A_{665}-2.69\times A_{645}$$

$$\text{Carotenoids }\left(\text{mg}/\text{liter}\right)\;=(1,000\times A_{470}-2.05\times \text{Chl }a)÷245$$

### Assay for photosynthesis.

To assay the photosynthetic response of the algal cells cultured under nutrient deficient conditions, the maximum quantum yield of photosystem (PS) II (Fv/Fm) and the relative electron transport rate (rETR) were investigated using fast chlorophyll fluorescence on a pulse-amplitude modulation fluorometer (XE-PAM; Walz, Effeltrich, Germany). Fv/Fm was measured after the algal cells were incubated in darkness for 15 min.

### Assay for alkaline phosphatase activity.

Alkaline phosphatase activity (APA) was determined by an APA assay kit (number P0321S; Beyotime Biotechnology, China). Algal cells were collected using centrifugation at 6,000 × *g* for 10 min and washed twice with PBS (0.01 M, pH 7.4). Cells were resuspended in PBS (1 ml) and sonicated on ice using an ultrasonic cell disruption system (Ningbo Scientz Biotechnological Co., Ltd., China) (80 W, 5 s:5 s, 100 times). The remaining debris was removed by centrifugation at 10,000 × *g* at 4°C for 10 min. The supernatant was taken for APA measurement according to the APA assay kit’s operation manual. APA was detected based on the principle that AP reacts with *para*-nitrophenyl phosphate to produce *para*-nitrophenol, which is a yellow substance and determined by absorbance at 405 nm.

### Algal lysis and lipid analysis of *P. donghaiense* treated with an algicidal bacterium.

The culture of the algicidal bacterium *Paracoccus* sp. Y42 and algal lysis assays were performed as described in our previous publication (57). The lipid contents and fatty acid compositions of *P. donghaiense* cultures separately treated with 1%, 3%, and 5% Y42 supernatant for 72 h were determined according to the methods described above. Algal cells in the control, 1% treatment, and 3% treatment groups were sonicated on ice using an ultrasonic cell disruption system (80 W, 5 s:5 s, 100 times) for lipid extraction. Algal cultures treated with 5% Y42 supernatant were directly used for lipid extraction without sonication.

### Statistical analysis.

All experiments in this study were carried out in triplicates, and the results are reported as the means ± standard deviations (SDs). The data were subjected to one-way analysis of variance (ANOVA) using the SPSS statistical package. *t* tests were conducted to determine the significance of differences. A *P* value of <0.05 was considered statistically significant.

## References

[1] Hossain N, Mahlia TMI. 2019. Progress in physicochemical parameters of microalgae cultivation for biofuel production. Crit Rev Biotechnol 39:835–859. 10.1080/07388551.2019.1624945. 31185749

[2] Borowitzka MA. 1995. Microalgae as sources of pharmaceuticals and other biologically active compounds. J Appl Phycol 7:3–15. 10.1007/BF00003544.

[3] Pulz O, Gross W. 2004. Valuable products from biotechnology of microalgae. Appl Microbiol Biotechnol 65:635–648. 10.1007/s00253-004-1647-x. 15300417

[4] Chisti Y. 2007. Biodiesel from microalgae. Biotechnol Adv 25:294–306. 10.1016/j.biotechadv.2007.02.001. 17350212

[5] Milano J, Ong HW, Masjuki HH, Chong WT, Lam MK, Loh PK, Vellayan V. 2016. Microalgae biofuels as an alternative to fossil fuel for power generation. Renew Sustain Energy Rev 58:180–197. 10.1016/j.rser.2015.12.150.

[6] Liu B, Benning C. 2013. Lipid metabolism in microalgae distinguishes itself. Curr Opin Biotechnol 24:300–309. 10.1016/j.copbio.2012.08.008. 22981869

[7] Li X, Přibyl P, Bišová K, Kawano S, Cepák V, Zachleder V, Čížková M, Brányiková I, Vítová M. 2013. The microalga *Parachlorella kessleri*-a novel highly efficient lipid producer. Biotechnol Bioeng 110:97–107. 10.1002/bit.24595. 22766749

[8] Li L, Cui J, Liu Q, Ding Y, Liu J. 2015. Screening and phylogenetic analysis of lipid-rich microalgae. Algal Res 11:381–386. 10.1016/j.algal.2015.02.028.

[9] Ho S-H, Nakanishi A, Ye X, Chang J-S, Hara K, Hasunuma T, Kondo A. 2014. Optimizing biodiesel production in marine *Chlamydomonas* sp. JSC4 through metabolic profiling and an innovative salinity-gradient strategy. Biotechnol Biofuels 7:97. 10.1186/1754-6834-7-97. 25002905 PMC4079173

[10] Scranton MA, Ostrand JT, Fields FJ, Mayfield SP. 2015. *Chlamydomonas* as a model for biofuels and bio-products production. Plant J 82:523–531. 10.1111/tpj.12780. 25641390 PMC5531182

[11] Cho K, Hur SP, Lee CH, Ko K, Lee YJ, Kim KN, Kim MS, Chung YH, Kim D, Oda T. 2016. Bioflocculation of the oceanic microalga *Dunaliella salina* by the bloom-forming dinoflagellate *Heterocapsa circularisquama*, and its effect on biodiesel properties of the biomass. Bioresour Technol 202:257–261. 10.1016/j.biortech.2015.12.047. 26733439

[12] Ma Y, Wang Z, Yu C, Yin Y, Zhou G. 2014. Evaluation of the potential of 9 *Nannochloropsis* strains for biodiesel production. Bioresour Technol 167:503–509. 10.1016/j.biortech.2014.06.047. 25013933

[13] Xiao Y, Zhang J, Cui J, Feng Y, Cui Q. 2013. Metabolic profiles of *Nannochloropsis oceanica* IMET1 under nitrogen-deficiency stress. Bioresour Technol 130:731–738. 10.1016/j.biortech.2012.11.116. 23334034

[14] Xu H, Miao X, Wu Q. 2006. High quality biodiesel production from a microalga *Chlorella protothecoides* by heterotrophic growth in fermenters. J Biotechnol 126:499–507. 10.1016/j.jbiotec.2006.05.002. 16772097

[15] Zhang W, Zhao Y, Cui B, Wang H, Liu T. 2016. Evaluation of filamentous green algae as feedstocks for biofuel production. Bioresour Technol 220:407–413. 10.1016/j.biortech.2016.08.106. 27598569

[16] Wu HQ, Miao XL. 2014. Biodiesel quality and biochemical changes of microalgae *Chlorella pyrenoidosa* and *Scenedesmus obliquus* in response to nitrate levels. Bioresour Technol 170:421–427. 10.1016/j.biortech.2014.08.017. 25164333

[17] Xu D, Gao Z, Li F, Fan X, Zhang X, Ye N, Mou S, Liang C, Li D. 2013. Detection and quantitation of lipid in the microalga *Tetraselmis subcordiformis* (Wille) Butcher with BODIPY 505/515 staining. Bioresour Technol 127:386–390. 10.1016/j.biortech.2012.09.068. 23138061

[18] Hildebrand M, Davis AK, Smith SR, Traller JC, Abbriano R. 2012. The place of diatoms in the biofuel industry. Biofuel 3:221–240. 10.4155/bfs.11.157.

[19] Song M, Pei H, Hu W, Ma G. 2013. Evaluation of the potential of 10 microalgal strains for biodiesel production. Bioresour Technol 141:245–251. 10.1016/j.biortech.2013.02.024. 23489572

[20] Oh SH, Han JG, Kim Y, Ha JH, Kim SS, Jeong MH, Jeong HS, Kim NY, Cho JS, Yoon WB, Lee SY, Kang DH, Lee HY. 2009. Lipid production in *Porphyridium cruentum* grown under different culture conditions. J Biosci Bioeng 108:429–434. 10.1016/j.jbiosc.2009.05.020. 19804869

[21] Ganuza E, Izquierdo MS. 2007. Lipid accumulation in *Schizochytrium* G13/2S produced in continuous culture. Appl Microbiol Biotechnol 76:985–990. 10.1007/s00253-007-1019-4. 17694304

[22] Ma X, Chen T, Yang B, Liu J, Chen F. 1975. Lipid production from *Nannochloropsis*. Biochem Pharmacol 24:1639–1641. 10.3390/md14040061. 10

[23] Kanaga K, Pandey A, Kumar S, Geetanjali. 2016. Multi-objective optimization of media nutrients for enhanced production of algae biomass and fatty acid biosynthesis from *Chlorella pyrenoidosa* NCIM 2738. Bioresour Technol 200:940–950. 10.1016/j.biortech.2015.11.017. 26613206

[24] Kim S, Kim H, Ko D, Yamaoka Y, Otsuru M, Kawai-Yamada M, Ishikawa T, Oh HM, Nishida I, Li-Beisson Y, Lee Y. 2013. Rapid induction of lipid droplets in *Chlamydomonas reinhardtii* and *Chlorella vulgaris* by brefeldin A. PLoS One 8:e81978. 10.1371/journal.pone.0081978. 24349166 PMC3862487

[25] Li-Beisson Y, Thelen JJ, Fedosejevs E, Harwood JL. 2019. The lipid biochemistry of eukaryotic algae. Prog Lipid Res 74:31–68. 10.1016/j.plipres.2019.01.003. 30703388

[26] Wase N, Black P, DiRusso C. 2018. Innovations in improving lipid production: algal chemical genetics. Prog Lipid Res 71:101–123. 10.1016/j.plipres.2018.07.001. 30017715

[27] Conte M, Lupette J, Seddiki K, Mei C, Dolch LJ, Gros V, Barette C, Rébeillé F, Jouhet J, Maréchal E. 2018. Screening for biologically annotated drugs that trigger triacylglycerol accumulation in the diatom *Phaeodactylum*. Plant Physiol 177:532–552. 10.1104/pp.17.01804. 29535162 PMC6001342

[28] Gong Y, Jiang M. 2011. Biodiesel production with microalgae as feedstock: from strains to biodiesel. Biotechnol Lett 33:1269–1284. 10.1007/s10529-011-0574-z. 21380528

[29] Franz A, Danielewicz MA, Wong DM, Anderson LA, Boothe J. 2013. Phenotypic screening with oleaginous microalgae reveals modulators of lipid productivity. ACS Chem Biol 8:1053–1062. 10.1021/cb300573r. 23521767

[30] Shaikh KM, Nesamma AA, Abdin MZ, Jutur PP. 2018. Evaluation of growth and lipid profiles in six different microalgal strains for biofuel production. *In* Kumar S, Sani R, Yadav Y(ed), Conference Proceedings of the Second International Conference on Recent Advances in Bioenergy Research. Springer proceedings in energy. Springer, Singapore. 10.1007/978-981-10-6107-3_1.

[31] Grünewald CF. 2012. Dinoflagellates as feedstock for biodiesel production. *In* Gordon R, Seckbach J (ed), The science of algal fuels. Cellular origin, life in extreme habitats and astrobiology, vol 25. Springer Science, Dordrecht, Netherlands. 10.1007/978-94-007-5110-1_13.

[32] Calbet A, Bertos M, Fuentes-Grünewald C, Alacid E, Figueroa R, Renom B, Garcés E. 2011. Intraspecific variability in *Karlodinium veneficum*: growth rates, mixotrophy, and lipid composition. Harmful Algae 10:654–667. 10.1016/j.hal.2011.05.001.

[33] Chen C-Y, Yeh K-L, Aisyah R, Lee D-J, Chang J-S. 2011. Cultivation, photobioreactor design and harvesting of microalgae for biodiesel production: a critical review. Bioresour Technol 102:71–81. 10.1016/j.biortech.2010.06.159. 20674344

[34] de Boer K, Moheimani NR, Borowitzka MA, Bahri PA. 2012. Extraction and conversion pathways for microalgae to biodiesel: a review focused on energy consumption. J Appl Phycol 24:1681–1698. 10.1007/s10811-012-9835-z.

[35] Lenneman EM, Wang P, Barney BM. 2014. Potential application of algicidal bacteria for improved lipid recovery with specific algae. FEMS Microbiol Lett 354:102–110. 10.1111/1574-6968.12436. 24673371

[36] Chen CY, Bai MD, Chang JS. 2013. Improving microalgal oil collecting efficiency by pretreating the microalgal cell wall with destructive bacteria. Biochem Engineering J 81:170–176. 10.1016/j.bej.2013.10.014.

[37] Liu L, Zhou J, Zheng B, Cai W, Lin K, Tang J. 2013. Temporal and spatial distribution of red tide outbreaks in the Yangtze River Estuary and adjacent waters, China. Mar Pollut Bull 72:213–221. 10.1016/j.marpolbul.2013.04.002. 23628547

[38] Tang EPY. 1996. Why do dinoflagellates have lower growth rates? J Phycol 132:80–84. 10.1111/j.0022-3646.1996.00080.x.

[39] Ahmad AL, Mat Yasin NH, Derek CJC, Lim JK. 2011. Microalgae as a sustainable energy source for biodiesel production: a review. Renew Sustain Energy Rev 15:584–593. 10.1016/j.rser.2010.09.018.

[40] Zhukova NA, Aizdaicher NA. 1995. Fatty acid composition of 15 species of marine microalgae. Phytochem 39:351–356. 10.1016/0031-9422(94)00913-E.

[41] Crawford MA, Broadhurst CL. 2012. The role of docosahexaenoic and the marine food web as determinants of evolution and hominid brain development: the challenge for human sustainability. Nutr Health 21:17–39. 10.1177/0260106012437550. 22544773

[42] Cohen Z, Khozin-Goldberg I. 2010. Searching for polyunsaturated fatty acid-rich photosynthetic microalgae, p 201–224. *In* Cohen Z, Ratledge C (ed), Single cell oils. Microbial and algal oils, 2nd ed. Academic Press, Cambridge, MA.

[43] Chauton MS, Kjell IR, Niels HN, Ragnar T, Hans TK. 2015. A techno-economic analysis of industrial production of marine microalgae as a source of EPA and DHA-rich raw material for aquafeed: research challenges and possibilities. Aquaculture 436:95–103. 10.1016/j.aquaculture.2014.10.038.

[44] Arias-Peñaranda MT, Cristiani-Urbina E, Montes-Horcasitas C, Esparza-García F, Torzillo G, Cañizares-Villanueva RO. 2013. *Scenedesmus incrassatulus* CLHE-Si01: a potential source of renewable lipid for high quality biodiesel production. Bioresour Technol 140:158–164. 10.1016/j.biortech.2013.04.080. 23688667

[45] Francisco EC, Neves DB, Jacob-Lopes E, Franco TT. 2010. Microalgae as feedstock for biodiesel production: carbon dioxide sequestration, lipid production and biofuel quality. J Chem Technol Biotechnol 85:395–403. 10.1002/jctb.2338.

[46] Giakoumis EG. 2013. A statistical investigation of biodiesel physical and chemical properties, and their correlation with the degree of unsaturation. Renew Energy 50:858–878. 10.1016/j.renene.2012.07.040.

[47] Hockin NL, Mock T, Mulholland F, Kopriva S, Malin G. 2012. The response of diatom central carbon metabolism to nitrogen starvation is different from that of green algae and higher plants. Plant Physiol 158:299–312. 10.1104/pp.111.184333. 22065419 PMC3252072

[48] Lopez Garcia de Lomana A, Schauble S, Valenzuela J, Imam S, Carter W, Bilgin DD, Yohn CB, Turkarslan S, Reiss DJ, Orellana MV, Price ND, Baliga NS. 2015. Transcriptional program for nitrogen starvation-induced lipid accumulation in *Chlamydomonas reinhardtii*. Biotechnol Biofuels 8:207. 10.1186/s13068-015-0391-z. 26633994 PMC4667458

[49] Goncalves EC, Koh J, Zhu N, Yoo MJ, Chen S, Matsuo T, Johnson JV, Rathinasabapathi B. 2016. Nitrogen starvation-induced accumulation of triacylglycerol in the green algae: evidence for a role for ROC40, a transcription factor involved in circadian rhythm. Plant J 85:743–757. 10.1111/tpj.13144. 26920093

[50] Kokabi K, Gorelova O, Ismagulova T, Itkin M, Malitsky S, Boussiba S, Solovchenko A, Khozin-Goldberg I. 2019. Metabolomic foundation for differential responses of lipid metabolism to nitrogen and phosphorus in an arachidonic acid-producing green microalga. Plant Sci 283:95–115. 10.1016/j.plantsci.2019.02.008. 31128719

[51] Ou L, X Q, Shi X, Feng Q, Zhang S, Lu S, Qi Y. 2020. Alkaline phosphatase activities and regulation in three harmful *Prorocentrum* species from the coastal waters of the East China Sea. Microb Ecol 79:459–471. 10.1007/s00248-019-01399-3. 31267157

[52] Yao L, Shen H, Wang N, Tatlay J, Li L, Tan TW, Lee YK. 2017. Elevated acetyl-CoA by amino acid recycling fuels microalgal neutral lipid accumulation in exponential growth phase for biofuel production. Plant Biotechnol J 15:497–509. 10.1111/pbi.12648. 27734577 PMC5362678

[53] Chungjatupornchai W, Areerat K, Fa-Aroonsawat S. 2019. Increased triacylglycerol production in oleaginous microalga *Neochloris oleoabundans* by overexpression of plastidial lysophosphatidic acid acyltransferase. Microb Cell Fact 18:53. 10.1186/s12934-019-1104-2. 30866936 PMC6415348

[54] Mutlu YB, Isik O, Uslu Y, Koc K, Durmaz Y. 2011. The effects of nitrogen and phosphorus deficiencies and nitrite addition on the lipid content of *Chlorella vulgaris* (Chlorophyceae). Afr J Biotechnol 10:453–456.

[55] Guo FJ, Wang H, Wang JF, Zhou WJ, Gao LL, Chen L, Dong QZ, Zhang W, Liu TZ. 2014. Special biochemical responses to nitrogen deprivation of filamentous oleaginous microalgae *Tribonema* sp. Bioresour Technol 158:19–24. 10.1016/j.biortech.2014.01.144. 24583210

[56] Hu H, Wang HF, Ma LL, Shen XF, Zeng RJ. 2018. Effects of nitrogen and phosphorous stress on the formation of high value LC-PUFAs in *Porphyridium cruentum*. Appl Microbiol Biotechnol 102:5763–5773. 10.1007/s00253-018-8943-3. 29671003

[57] Zhang F, Ye Q, Chen Q, Yang K, Zhang D, Chen Z, Lu S, Shao X, Fan Y, Yao L, Ke L, Zheng T, Xu H. 2018. Algicidal activity of novel marine bacterium *Paracoccus* sp. strain Y42 against a harmful algal-bloom-causing dinoflagellate, *Prorocentrum donghaiense*. Appl Environ Microbiol 84:e01015-18. 10.1128/AEM.01015-18. 30054369 PMC6147001

[58] Ma YB, Wang ZY, Zhu M, Yu CJ, Cao YP, Zhang DY, Zhou GK. 2013. Increased lipid productivity and TAG content in *Nannochloropsis* by heavy-ion irradiation mutagenesis. Bioresour Technol 136:360–367. 10.1016/j.biortech.2013.03.020. 23567703

[59] Cho K, Lee CH, Ko K, Lee YJ, Kim KN, Kim MK, Chung YH, Kim D, Yeo I-K, Oda T. 2016. Use of phenol-induced oxidative stress acclimation to stimulate cell growth and biodiesel production by the oceanic microalga *Dunaliella salina*. Algal Res 17:61–66. 10.1016/j.algal.2016.04.023.

[60] Mandotra SK, Kumar P, Suseela MR, Ramteke PW. 2014. Fresh water green microalga *Scenedesmus abundans*: a potential feedstock for high quality biodiesel production. Bioresour Technol 156:42–47. 10.1016/j.biortech.2013.12.127. 24486936

